# Efficacy of hemostatic gel for perioperative bleeding and prevention of delayed bleeding of cold snare polypectomy under anticoagulant

**DOI:** 10.1002/jgh3.13029

**Published:** 2024-01-19

**Authors:** Naohisa Yoshida, Osamu Dohi, Yoshikazu Inagaki, Yuri Tomita, Hikaru Hashimoto, Reo Kobayashi, Ken Inoue, Ryohei Hirose, Yasutaka Morimoto, Yutaka Inada, Takaaki Murakami, Yoshito Itoh

**Affiliations:** ^1^ Department of Molecular Gastroenterology and Hepatology, Kyoto Prefectural University of Medicine Graduate School of Medical Science Kyoto Japan; ^2^ Department of Gastroenterology Nishijin Hospital Kyoto Japan; ^3^ Department of Gastroenterology Koseikai Takeda Hospital Kyoto Japan; ^4^ Department of Gastroenterology Osaka General Hospital of West Japan Railway Company Osaka Japan; ^5^ Department of Gastroenterology Kyoto Saiseikai Hospital Kyoto Japan; ^6^ Department of Gastroenterology Kyoto First Red Cross Hospital Kyoto Japan; ^7^ Department of Gastroenterology Aiseikai Yamashina Hospital Kyoto Japan

**Keywords:** cold snare polypectomy, colonoscopy, colorectal polyps, delayed bleeding, perioperative bleeding

## Abstract

**Background and Aim:**

A hemostatic gel, PuraStat (3‐D Matrix, Tokyo, Japan), is used for various gastrointestinal hemostasis. In this study, we analyzed the efficacy of PuraStat for perioperative bleeding (POB) and prevention of delayed bleeding (DB) to colorectal cold snare polypectomy (CSP) with continuous anticoagulant.

**Methods:**

This was a single‐center, retrospective study. Subjects were lesions of 2–9 mm under continuous anticoagulant from 2021 to 2023 and treated with PuraStat for POB. The definition of POB was bleeding which did not stop spontaneously by 1.0–1.5 min after resection and needed hemostasis. Successful hemostasis was defined as cessation of bleeding within 1.0–1.5 min after spraying PuraStat and the rate of it and risk factors of POB were analyzed. For comparison, cases receiving previous CSP without PuraStat were extracted from all cases with CSP (2018‐2021), and POB and DB rate (DBR) were analyzed after propensity score matching.

**Results:**

One hundred twenty‐two lesions (91: direct oral anticoagulant (DOAC), 31: warfarin) with anticoagulant were analyzed and the rate of successful hemostasis with PuraStat was 92.6% (DOAC/warfarin: 93.4%/80.6%, *P* = 0.01). The rate of DB was 0.0%. Multivariate analysis showed that significant risk factors about unsuccessful hemostasis for POB with PuraStat were lesion size 8–9 mm (*P* < 0.01), warfarin (*P* = 0.01), and combination of antiplatelet (*P* = 0.01). Regarding the comparison about CSP with/without PuraStat, the clipping rate and DBR were 8.5%/94.9% (*P* < 0.01) and 0%/1.7% (*P* = 1.0).

**Conclusion:**

The effects of PuraStat for POB and DB in colorectal CSP with continuous anticoagulant were acceptable.

## Graphical abstract

In this study, we analyzed 122 cases with colorectal cold snare polypectomy (CSP) with continuous anticoagulant (91 cases: direct oral anticoagulant, 31 cases: warfarin) treated with a hemostatic gel, PuraStat. The rates of successful hemostasis and delayed bleeding were 92.6% and 0.0%, respectively. The rate of clipping after CSP was reduced to 7.4%.

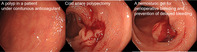

## Introduction

Polyp resection has been reported to lead to a reduction in colorectal cancer deaths.^1^ Several guidelines show that cold snare polypectomy (CSP) is a useful and safe technique for resecting polyps <10 mm.^2^, ^3^, ^4^ A study of benign lesions <10 mm in patients on warfarin showed a significant difference in delayed bleeding rate (DBR) between CSP and hot polypectomy (0% *vs* 14%, *P* = 0.027).^5^ Two large studies showed that the DBR of CSP was 0.14% (26/18007) and 0.51% (11/4433).^6^, ^7^ Significant risk factors for DB (odds ratio: 95% confidence interval [CI]) were anticoagulant (7.866: 2.063–29.988), antiplatelet (4.521: 1.817–11.249), rectal location (3.674: 1.426–9.465), lesion size ≥5 mm (3.251: 1.417–7.463), and polypoid morphology (7.087: 2.081–24.132).^6^ Especially regarding anticoagulant, a Japanese large‐scale randomized control trial (RCT) showed that DBR in CSP with continuous anticoagulant was high at 4.7% (95% CI: 0.2–9.2).^8^

PureStat is an absorbent local hemostatic agent for oozing from various organs, and it has been covered by Japanese insurance since 2021 as hemostatic agent for oozing from various gastrointestinal tracts.^9^, ^10^ Amino acid peptides aggregate to form peptide nanofibers and hydrogel when in contact with body fluids and hydrogel coats bleeding point for hemostasis.^11^ Various reports have demonstrated the efficacy of PuraStat for perioperative bleeding (POB) in esophageal/gastric/duodenal endoscopic mucosal resection (EMR) and CSP, esophageal/gastric/colorectal endoscopic submucosal dissection (ESD), endoscopic papillectomy, and acute upper/lower gastrointestinal bleeding.^11^, ^12^, ^13^, ^14^, ^15^, ^16^, ^17^, ^18^, ^19^, ^20^

Endoscopic clipping is generally performed to treat POB and prevent DB of colorectal CSP in cases with anticoagulant. However, multiple clippings are sometimes required, and accurate clipping is difficult in some locations and situations. In addition, DB can occur even when clipping is performed. We hypothesized that PuraStat can be easy to apply compared with clipping and quickly covers the entire CSP ulcer. To our knowledge, there are no reports of PuraStat about the efficacy in colorectal CSP. In this study, we investigated the effect of PuraStat for POB and prevention of DB for colorectal CSP in continuous anticoagulant.

## Methods

**Figure 1:**
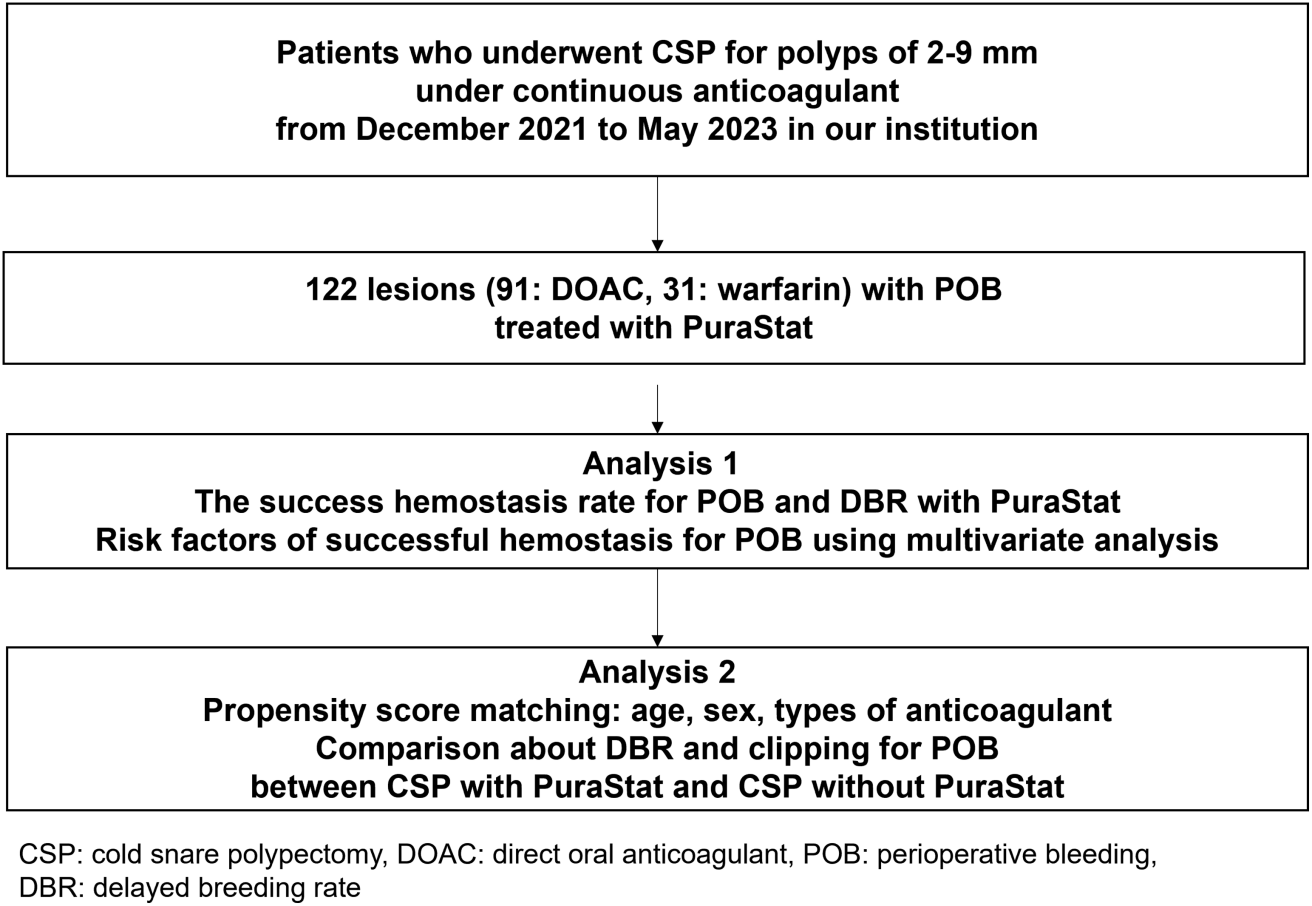
A flow diagram of the current study.

This was a single‐center, retrospective cohort study. Consequent patients undergoing CSP for polyps of 2–9 mm under continuous anticoagulant (direct oral anticoagulant (DOAC) and warfarin) due to high risk of thromboembolism from December 2021 to May 2023 in our institution were reviewed (Fig. 1). High risk of thromboembolism associated with withdrawal of anticoagulants was according to a Japanese guideline as follows: (i) history of cardiogenic brain embolism, (ii) atrial fibrillation accompanying valvular heart disease, (iii) atrial fibrillation without valvular heart disease but with high risk of stroke, (iv) following mechanical mitral valve replacement, (v) history of thromboembolism following mechanical valve replacement, (vi) antiphospholipid antibody syndrome, and (vii) deep vein thrombosis/pulmonary thromboembolism.^21^ Cases with POB treated with PuraStat were extracted and analyzed regarding POB and DB.

The primary outcome of this study was to evaluate the efficacy of PuraStat in preventing DB after CSP. The secondary outcome was to evaluate the efficacy of PuraStat for hemostasis of POB. Risk factors for POB in these cases were also examined. In addition, CSP with PuraStat and without PuraStat were compared for DBR and clipping for POB. Two analyses were performed to examine these outcomes.

**Figure 2:**
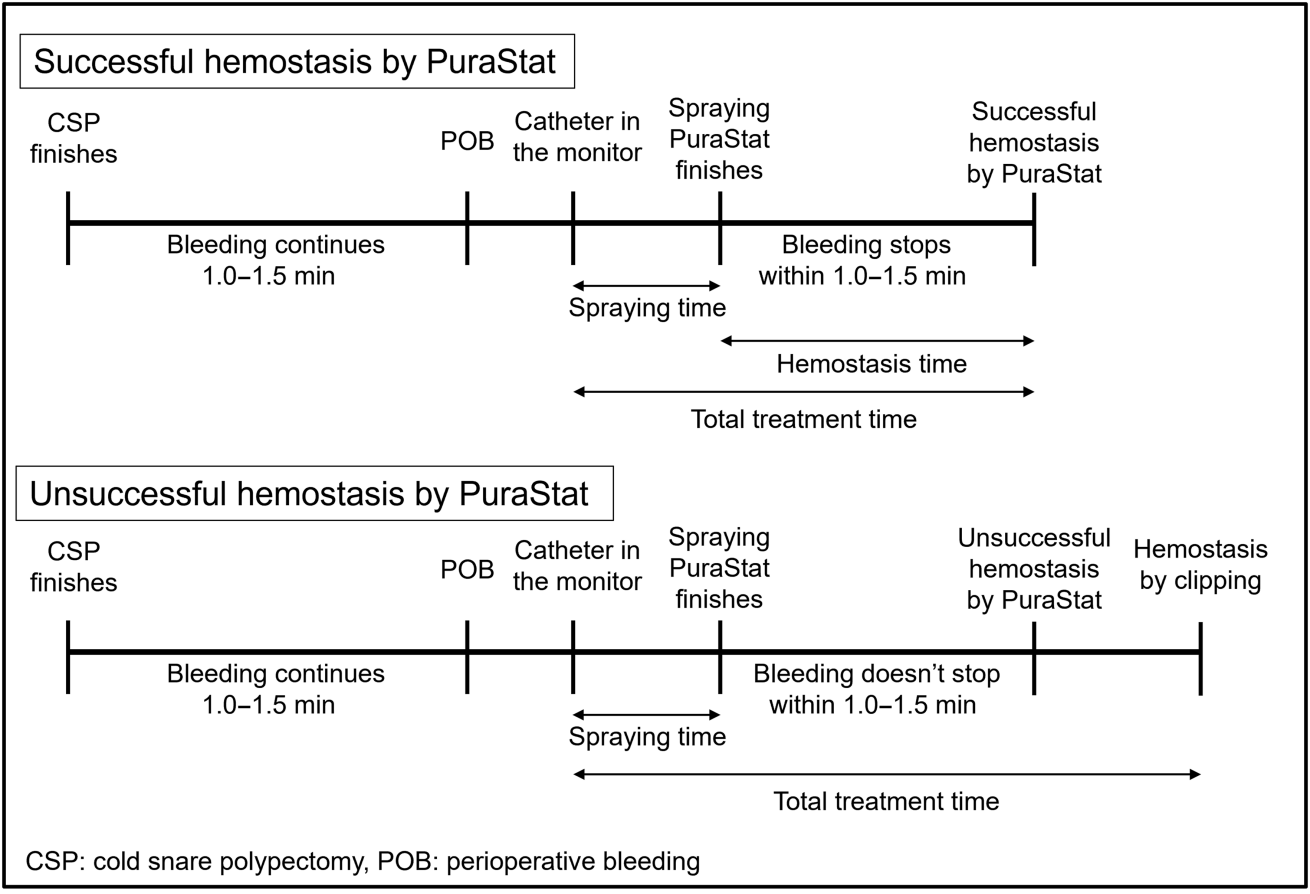
Treatment progress of lesions with successful or unsuccessful hemostasis by PuraStat including the definition of spraying time, hemostasis time, and total treatment time.

In Analysis 1, the rates of successful hemostasis for POB with PuraStat in overall anticoagulant, DOAC, and warfarin were analyzed. The DBRs of each drug were also examined. The definition of POB was bleeding which did not stop spontaneously by 1.0–1.5 min after resection and needed hemostasis. The successful hemostasis was defined as the complete cessation of bleeding within 1.0–1.5 min after spraying PuraStat. The time for spraying PuraStat was calculated from the time the catheter appeared on the monitor to the time PuraStat was finished spraying appropriately (Fig. 2). Hemostasis time was calculated from the time PuraStat was finished spraying to the time hemostasis was achieved only in lesions achieving successful hemostasis by PuraStat. Additionally, total treatment time for POB (from spraying to hemostasis) in lesions both with successful and unsuccessful hemostasis by PuraStat. In cases with unsuccessful hemostasis, the time of additional clipping included the total treatment time. Risk factors for successful hemostasis for CSP with anticoagulant treated with PuraStat were also analyzed, regarding age, sex, types of anticoagulant, types of DOAC (only for lesions with DOAC), prothrombin time‐international normalized ratio (PT‐INR) (only for lesions with warfarin), prescription of steroid, hemodialysis, combination of antiplatelet, lesion size, location, morphology, en bloc resection, histopathological complete resection, and histopathology.

In Analysis 2, among all cases in Analysis 1, previous CSP without PuraStat from January 2018 to December 2021, when PuraStat was not released in Japan were extracted and lesions with detailed information and recorded videos were examined as a comparison (Fig. 1). In this period, the POB which did not stop spontaneously by about 1.0–1.5 min was treated with clipping according to each endoscopist's decision. Clipping was also performed to prevent DB according to each endoscopist's decision. According to endoscopic figures or movies, we confirmed whether clipping after CSP was performed. To minimize the bias of patient characteristics between CSP with PuraStat and CSP without PuraStat, propensity score matching was performed about age, sex, and type of anticoagulant. The clipping rate after CSP between CSP with PuraStat and CSP without PuraStat was compared. In the CSP without PuraStat group, various characteristics, therapeutic results, rate of clipping, total treatment time for POB, and DBR were analyzed according to medical records and endoscopic images/movies. Total treatment time of clipping was calculated from the time the clip device was shown in the monitor to achievement of complete cessation of bleeding by one to several clips.

### CSP and use of PuraStat for POB

**Figure 3:**
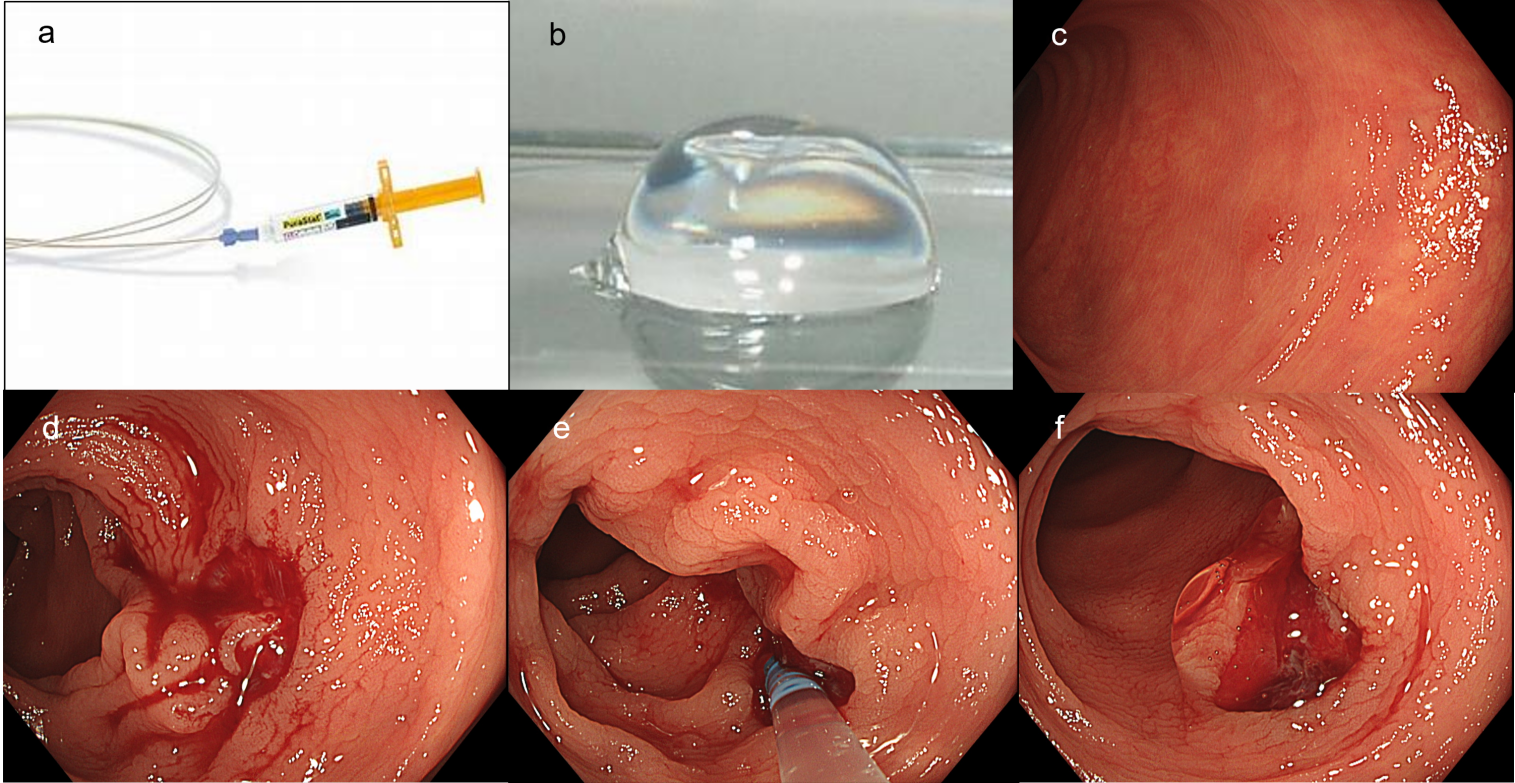
Case presentation of CSP with PuraStat. (a) PuraStat, 3‐mL formulation. (b) Gel of PuraStat. (c) 73‐year‐old male, edoxaban, a non‐polypoid lesion, 5‐mm, sigmoid colon, low‐grade adenoma. (d) CSP was performed. (e) The catheter was gently placed on an ulcer for spraying PuraStat. (f) PuraStat gel covered the ulcer and the amount of PuraStat was 0.2 mL. Hemostasis was achieved in 30 s.

A variety of snare types used in this study were Captivator Cold 9 mm (Boston Scientific, USA), Exacto 9 mm (US Endoscopy, USA), and Cold snare 10 and 15 mm (Micro‐Tech Co., China). PuraStat 1.0‐ or 3.0‐mL formulations were used depending on the estimated number of polyps. CSP was performed for lesions of 2–9 mm and PuraStat was sprayed to an ulcer caused by CSP with a catheter (TOP, Co. Tokyo, Japan) when POB occurred (Fig. 3, Video S1). In cases that bleeding point was found accurately, the catheter was gently pushed to the point and 0.2–0.4 mL of PuraSta was sprayed. In cases that bleeding point was unclear, the catheter was gently placed on an ulcer and PuraStat was sprayed.^15^ The maximum amount of PuraStat was fixed at 0.4 mL for each lesion.

All colonoscopies were performed by 10 endoscopists (five experts and five nonexperts). Five experts had experience in performing more than 5000 colonoscopies. Regarding endoscopic morphology, all lesions were classified as polypoid or non‐polypoid.^22^ Histopathological diagnosis of lesions, including adenoma and SSL, was made according to the 2019 WHO classification.^23^ The histopathological complete resection was defined as negative horizontal and vertical margin of lesions.

This study was a subgroup analysis of another study on endoscopic diagnosis and treatment approved by the Ethics Committee of the Kyoto Prefectural University of Medicine (ERB‐C‐1704‐3) and was confirmed and approved by each institution. This was a retrospective study and patients' informed consent was obtained with the option opt‐out from participating. This study was conducted in accordance with the Declaration of Helsinki of the World Medical Association.

### Statistical analyses

In our pilot study of 30 lesions with CSP under continuous either anticoagulant or antiplatelet treated with PuraStat, the DBR of CSP was 0.0% (not published). We hypothesized that the DBR of CSP with PuraStat was 0.0% for CSP with anticoagulant in this large‐scale study. The rate of DBR in a previous RCT under CSP with continuous anticoagulant in Japan was 4.7%.^8^ We considered that the upper limit of the 95% CI for the DBR with PuraStat should be less than 4.7%. Therefore, the sample size was calculated as 95 using the Agresti‐Coull test. Taking into account the 10% of excluded cases with incomplete information, at least 105 cases were needed for this study. The Mann–Whitney *U* test was used to compare continuous variables. Categorical variables were analyzed using chi‐squared test and Yates continuity correction. In propensity score matching, the absolute standardized difference (ASD) value was examined to assess the validity of the matching, and an ASD value of ≤0.2 was determined to be appropriate. All statistical analyses were performed with SPSS software (IBM Japan, Ltd., Tokyo, Japan). *P* < 0.05 was considered significant for all statistical analyses.

## Results

In the study period, we reviewed 127 lesions in 40 patients receiving CSP under continuous anticoagulant. Among them, 122 lesions in 37 patients with POB were treated with PuraStat (Table 1, Fig. 1). The number of lesions of DOAC and warfarin were 91 and 31, respectively. Regarding the types of DOAC for lesions with DOAC, the number of edoxaban, rivaroxaban, apixaban, and dabigatran were 56, 22, 7, and 6. The PT‐INR for lesions with warfarin (mean ± SD) was 2.23 ± 0.46. The rate of combination of antiplatelet was 11.5%, and all of antiplatelet agents were continued. The tumor size (mm, mean ± SD) was 3.9 ± 2.3. The number of right‐sided colon, left‐sided colon, and rectum were 85/33/4. The rates of en bloc resection and complete resection were 97.5% and 63.1%, respectively.

**Table 1:** Patients' characteristics

Lesion number	122
Patient number	37
Age, mean ± SD (range)	74.0 ± 7.1 (54–85)
Sex, *n* (%), male/female	28/9 (75.7/24.3)
Antithrombotic drugs, *n* (%)	91/31
DOAC/warfarin, *n* (%)	(74.6/25.4)
Types of DOAC, *n* (%)	56/22/7/6
Edoxaban/rivaroxaban/apixaban/dabigatran	(61.5/24.2/7.7/6.6)
PT‐INR (for lesions with warfarin), mean ± SD	2.23 ± 0.46
Steroid, *n* (%)	21 (17.2)
Hemodialysis, *n* (%)	2 (1.6)
Combination of antiplatelet, *n* (%)	14 (11.5)
Tumor size, mm, mean ± SD (range)	3.9 ± 2.3 (2–9)
Tumor size of 8–9 mm	15 (12.3)
Location, *n* (%), colon/rectum	115/7 (94.3/5.7)
Morphology, % (*n*), polypoid/non‐polypoid	94/28 (77.0/23.0)
Insertion time, min, mean ± SD	6.8 ± 3.8
Total procedure time, min, mean ± SD	24.3 ± 7.1
En bloc resection, *n* (%)	119 (97.5)
Histopathological complete resection, *n* (%)	77 (63.1)
Histopathology, *n* (%), adenoma/SSL + HP	100/22 (82.0/18.0)

DOAC, direct oral anticoagulant; HP, hyperplastic polyp; PT‐INR, prothrombin time‐international normalized ratio; SSL, sessile serrated lesions.

The rate of successful hemostasis for POB in lesions with overall anticoagulant was 92.6% (95% CI: 86.4–96.2) (Table 2). Those in DOAC and warfarin were 93.4% (95% CI: 90.3–99.2) and 80.6% (95% CI: 63.3–91.1), respectively (*P* = 0.01). The time to spray PuraStat (seconds, mean ± SD) was 12.0 ± 4.0. The time to achieve hemostasis (seconds, mean ± SD) in 113 cases with successful hemostasis with PuraStat was 46.1 ± 27.0, and the rate of time ≤60 s was 77.0% (87/113). All lesions without successful hemostasis were treated with endoscopic clipping and hemostasis was achieved. The rate of endoscopic clipping was 7.4%. Total treatment time for POB (seconds, mean ± SD) was 65.8 ± 25.0. After CSP, the DBR for anticoagulant was 0.0% (95% CI: 0.0–3.7).

**Table 2:** Successful hemostasis for perioperative bleeding with PuraStat

	Successful hemostasis	DBR
Anticoagulant, % (95% CI) (*n*)	92.6 (86.4–96.2) (113/122)	0.0 (0.0–3.7) (0/122)
DOAC, % (95% CI) (*n*)	93.4* (90.3–99.2) (88/91)	0.0 (0.0–4.9) (0/91)
Warfarin, % (95% CI) (*n*)	80.6** (63.3–91.1) (25/31)	0.0 (0.0–13.1) (0/31)

* *vs* **: *P* = 0.01.

CI, confidence interval; DBR, delayed bleeding rate; DOAC, direct oral anticoagulant.

A comparison was made between successful and unsuccessful hemostasis (Table 3). Multivariate analysis (OR: 95% CI) showed that there were significant differences regarding type of anticoagulant: warfarin (13.8: 1.6–112.0, *P* = 0.01), combination of antiplatelet (18.8: 1.7–206.0, *P* = 0.01), and tumor size of 8–9 mm (28.9: 4.8–325.0, *P* < 0.01). Considering only lesions treated with warfarin, univariate analysis showed that there was a significant difference in mean PT‐INR between lesions with successful hemostasis and lesions with unsuccessful hemostasis (1.96 ± 0.33 and 2.82 ± 0.13, *P* < 0.01).

**Table 3:** Risk factors of successful hemostasis for POB in cases with anticoagulant

	Univariate analysis			Multivariate analysis		
	Successful hemostasis	Unsuccessful hemostasis	*P* value	OR	95% CI	*P* value
Lesion number, *n* (%)	113 (92.6)	9 (7.4)	—			
Age, mean ± SD	68.5 ± 13.1	74.3 ± 7.8	0.11			
Sex (male/female)	25/6 (80.6/19.4)	3/3 (50.0/50.0)	0.27			
Antithrombotic drugs, *n* (%), DOAC/warfarin	88/25 (77.9/22.1)	3/6 (33.3/66.7)	<0.01	13.8	1.6–112.0	0.01
Types of DOAC, *n* (%) Edoxaban/rivaroxaban/apixaban/dabigatran	53/22/7/6 (60.2/25.0/8.0/6.8)	3/0/0/0 (100.0/0/0/0)		n.c.		
PT‐INR: (for lesions with warfarin), mean ± SD	1.96 ± 0.33	2.82 ± 0.13	<0.01	n.c.		
Steroid, *n* (%)	20 (17.7)	1 (11.1)	0.96			
Hemodialysis, *n* (%)	0 (0.0)	2 (22.2)	<0.01	n.c.		
Combination of antiplatelet, *n* (%)	10 (8.8)	4 (44.4)	<0.01	18.8	1.7–206.0	0.01
Tumor size, mm, mean ± SD	3.5 ± 2.0	6.5 ± 2.8	<0.01			
Tumor size, 8–9 mm, *n* (%)	9 (8.0)	6 (66.7)	<0.01	28.9	4.8–325.0	<0.01
Location, *n* (%), colon/rectum	107/6 (94.7/5.3)	8/1 (88.9/11.1)	0.98			
Morphology, *n* (%), polypoid/non‐polypoid	87/26 (77.0/23.0)	7/2 (88.9/11.1)	0.72			
En bloc resection, *n* (%)	0 (0.0)	3 (33.3)	<0.01	n.c.		
Histopathological complete resection, *n* (%)	73 (65.5)	4 (44.4)	0.39	n.c.		
Histopathology, *n* (%), adenoma/SSL + HP	93/20 (82.3/17.7)	7/2 (77.8/22.2)	0.91			

CI, confidence interval; DOAC, direct oral anticoagulant; HP, hyperplastic polyp; OR, odds ratio; POB, perioperative bleeding; SSL, sessile serrated lesions.

There were nine lesions with unsuccessful hemostasis (Table 4). In lesions with DOAC, edoxaban was prescribed in three lesions. In six lesions with warfarin, PT‐INR ranged from 2.59 to 2.81. On the other hand, six lesions were larger than 8 mm.

**Table 4:** Lesions with unsuccessful hemostasis of POB

Lesions no.	Age	Sex	Drugs	Antiplatelet	PT‐INR	Others	Mor	Site	Size	Resection	Histopathology
1	79	F	Warfarin	Prasugrel	2.81		IIa	C	3	E	LGA
2			Warfarin	Prasugrel	2.81		IIa	A	9	P	SSL
3			Warfarin	Prasugrel	2.81		IIa	A	9	P	SSL
4	70	F	Warfarin		2.97	HD	Is	D	8	E	LGA
5			Warfarin		2.97	HD	Is	S	5	E	LGA
6	83	M	Warfarin		2.59		Is	R	9	E	LGA
7	74	M	DOAC Edoxaban	Aspirin			Is	C	6	E	LGA
8	54	M	DOAC Edoxaban				Is	A	8	E	LGA
9	74	F	DOAC Edoxaban			Steroid	Is	A	9	E	LGA

A, ascending colon; C, cecum; D, descending colon; E, en bloc resection; F, female; HD, hemodialysis; LGA, low‐grade adenoma; M, male; Mor, morphology; P, piecemeal resection; POB, perioperative hemorrhage; PT‐INR, prothrombin time‐international normalized ratio; R, rectum; S, sigmoid colon; SSL, sessile serrated lesions.

After propensity score matching about age, sex, and type of anticoagulant for CSP with PuraStat and CSP without PuraStat, ASD values of these factors were confirmed to be ≤0.2. Finally, 59 lesions in the two groups were matched. For POB, the clipping rate after CSP was 8.5% and 94.9% (*P* < 0.01) (Table 5). The multiple clipping was performed in 18 out of 56 lesions (26.8%) in the CSP without PuraStat group. Total treatment time for POB (seconds, mean ± SD) was 60.1 ± 27.0 and 75.0 ± 40.2 (*P* = 0.04). The DBR was 0% and 1.7% (*P* = 1.0). Regarding the case of DBR in the CSP without PuraStat group, a 75‐year‐old man on warfarin had a 9‐mm polypoid lesion in the sigmoid colon. The PT‐INR was 2.21. CSP was performed with two prophylactic clips for POB. DB occurred 3 days after CSP. Additional clipping was performed for DB and the PT‐INR was 2.50 at hemostasis.

**Table 5:** Comparison of clipping rate and DBR between CSP with PuraStat and CSP without PuraStat using propensity score matching

	Before matching			After matching		
	CSP with PuraStat	CSP without PuraStat	*P* value	CSP with PuraStat	CSP without PuraStat	*P* value
Lesion number	122	59	—	59	59	—
Patient number	37	19	—	22	19	—
Age, mean ± SD (range)	74.0 ± 7.1 (54–85)	73.0 ± 8.8 (46–85)	0.75	73.2 ± 7.2 (54–85)	73.0 ± 8.8 (46–85)	0.86
Sex, *n* (%), male/female	28/9 (75.7/24.3)	13/6 (75.5/24.5)	0.79	16/6 (72.2/27.3)	13/6 (75.5/24.5)	0.96
Antithrombotic drugs, *n* (%), warfarin/DOAC	91/31 (74.6/25.4)	37/22 (62.7/37.3)	0.09	36/23 (61.0/39.0)	37/22 (62.7/37.3)	0.85
Steroid, *n* (%)	21 (17.2)	6 (10.2)	0.21	7 (11.9)	6 (10.2)	1.0
Hemodialysis, *n* (%)	2 (1.6)	1 (1.7)	0.55	1 (1.7)	1 (1.7)	1.0
Combination of antiplatelet	14 (11.5)	8 (13.6)	0.69	8 (13.6)	8 (13.6)	1.0
Tumor size, mm, mean ± SD (range)	3.9 ± 2.3 (2–10)	4.0 ± 2.8 (2–10)	0.77	4.0 ± 2.3 (2–9)	4.0 ± 2.8 (2–9)	0.61
Location, *n* (%), colon/rectum	115/7 (94.3/5.7)	53/6 (84.5/15.5)	0.43	57/2 (96.6/3.7)	53/6 (84.5/15.5)	0.27
Morphology, *n* (%), polypoid/non‐polypoid	94/28 (77.0/23.0)	47/12 (77.4/22.6)	0.69	43/16 (72.9/27.1)	47/12 (77.4/22.6)	0.39
En bloc resection, % (*n*)	119 (97.5)	55 (97.4)	0.31	55 (97.4)	55 (97.4)	1.0
Clipping after CSP, *n* (%)	9 (7.4)	56 (94.9)	<0.01	5 (8.5)	59 (94.9)	<0.01
Treatment time for POB, secs, mean ± SD	65.8 ± 30.0	75.0 ± 40.2	0.04	60.1 ± 27.0	75.0 ± 40.2	0.04
Total procedure time, min mean ± SD	24.0 ± 6.1	27.8 ± 9.0	0.06	23.3 ± 6.0	27.8 ± 9.0	0.05
Histopathological complete resection, % (*n*)	77 (63.1)	40 (67.7)	0.54	38 (64.4)	40 (67.7)	0.70
Histopathology, % (*n*), adenoma/SSL + HP	100/22 (82.0/18.0)	50/9 (81.9/18.1)	0.64	49/10 (83.1/16.9)	50/9 (81.9/18.1)	0.80
DBR, *n* (%)	0 (0)	1 (1.7)	0.71	0 (0)	1 (1.7)	1.0

CSP, cold snare polypectomy; DBR, delayed bleeding rate; DOAC, direct oral anticoagulant; HP, hyperplastic polyp; PT‐INR, prothrombin time‐international normalized ratio; SSL, sessile serrated lesions.

## Discussion

In the current study, we demonstrated the efficacy of PuraStat. The rate of successful hemostasis for POB with PuraStat was 93.4% for lesions with DOAC and 80.6% for lesions with warfarin. There was a significant difference between the two groups. The rate of DBR for lesions with anticoagulant was 0.0% (95% CI: 0.0–3.7). Compared with CSP without PuraStat, CSP with PuraStat had a possibility to decrease the number of clips under cases with anticoagulant. This was the first report demonstrating the efficacy of PuraStat for POB and DB of colorectal CSP.

Regarding the efficacy of PuraStat in gastrointestinal bleeding, a recent multicenter study showed that the rate of successful hemostasis was 94% in 111 patients with acute upper and lower gastrointestinal (GI) bleeding.^17^ An RCT of esophageal/colorectal ESD showed a significant reduction in the use of thermal therapy for perioperative bleeding (POB) in the PuraStat group compared with the control group (49.3% *vs* 99.6%, *P* < 0.001).^15^ In 100 GI endoscopic resections (48 esophageal, 31 colorectal, 11 gastric and 10 duodenal procedures), PuraStat achieved successful hemostasis in 75% of POB and the DBR was low (3%).^14^ In the current study, we demonstrated the efficacy of hemostasis of POB and prevention of DB in colorectal CSP in patients receiving anticoagulant. The rates for success of hemostasis with PuraStat were 93.4% for DOAC and 80.6% for warfarin, and we were able to skip clipping in these cases. However, the rate of lesions with warfarin was not sufficient. We showed that warfarin, antiplatelet combination, and lesion size of 8–9 mm were risk factors for successful hemostasis of POB in PuraStat. Especially in lesions with warfarin, higher PT‐INR was a possible risk factor. In these cases, we thought that clipping could be a first‐line treatment instead of PuraStat.

Previously, we analyzed DB after colorectal CSP.^6^ DB occurred 0–5 days after CSP with an average of 1.6 ± 1.3 days. On the other hand, we previously reported that DB occurred 1–10 days after EMR with an average of 5.0 ± 3.6 days in patients taking DOACs.^24^ Furthermore, we reported that the mean time of onset of DB after colorectal ESD in cases with antithrombotic drugs was 7.5 ± 3.7 days.^25^ Compared with EMR and ESD, CSP is performed without electrocautery and causes less damage to deeper submucosal layers involving more large vessels.^26^ This may result in the relatively early onset of DB after CSP, even in patient taking antithrombotic drugs. In the current study, DBR was 0.0% (95% CI: 0.0–3.7%) in lesions treated with anticoagulant using PuraStat. The upper margin of 3.7% was lower than that of CSP with anticoagulant without PuraStat, 4.7% in the previous study.^8^ This suggested that PuraStat was effective in preventing DB, although there was no significant difference in DBR between CSP with PuraStat and CSP without PuraStat in the current study due to small sample size.

There are several reports demonstrating the efficacy of wound healing after endoscopic resection with PuraStat.^13^, ^15^ One report analyzed 53 gastric ESD with PuraStat on the ulcer after ESD and the DBR was 2.0%.^13^ The complete healing stage at 1 week was achieved in 96%, and the scar stage at 4/8 weeks was achieved in 19%/98%. These were suggested to be better than normal healing without PuraStat. Another report showed that the rate of complete wound healing after esophageal/colorectal ESD at 4 weeks was better in the PuraStat group than in the control group (48.8% *vs* 25.0%, *P* = 0.02).^15^ In the current study, the DBR was 0% for lesions with anticoagulant. We considered that this might be due to the wound healing efficacy of PuraStat. Endoscopic clipping after CSP under anticoagulant was performed for both treatment of POB and prevention of DB. According to our results, we suggest PuraStat can decrease the number of clipping due to it is efficacy about POB and DB.

Total treatment time for POB was significantly shorter in CSP with PuraStat than CSP without PuraStat though the difference was only 15 s on average. We considered that clipping needed more accurate techniques for deploying appropriate spot than PuraStat. Additionally, multiple clipping was performed in 26.8%. Thus, we considered that PuraStat emerges as a potentially time‐saving option for the treatment of POB, though total procedure time was not significant between two groups due to small sample size.

Regarding the cost benefit about PuraStat, the cost of this agent is 3 mL (39 600 yen: USD 273). On the other hand, endoscopic disposable clipping costs 3000–10 000 yen (USD: 20–69) for one piece. Thus, PuraStat is more expensive than clipping. However, we could show that a maximum of 0.4 mL of PuraStat was enough for POB in a lesion. At least seven lesions in one case could be treated with a 3‐mL formulation of PuraStat. According to the cost, PuraStat should be used in cases with multiple lesions without analyzed identified risk factors, compared with the cost of clipping in each area. On the other hand, the insurance coverage of PuraStat and clipping is different in each country. In Japan, PuraStat is covered by Japanese insurance reimbursement in addition to the CSP fee. Patients ≤69 years old, 70–74 years old, and ≥75 years old pay 30%, 20%, and 10% of the fee. And the rest of the fee is covered by the health insurance system. Conversely, endoscopic clipping is not covered by Japanese insurance reimbursement. Thus, the fee of clipping is included in the fee of CSP, and the fee is paid by each hospital. We believe that the use of PuraStat and clipping may be related to these conditions.

Regarding limitation, this study was a single‐center study with a small number of cases. The risk of thromboembolism and the continuous of antithrombotic drugs were assessed by each physician. A variety of snare types were used according to each physician's preference. Endoscopic clipping in the CSP without PuraStat group was decided by each physician. In addition, the reason for the clipping, to treat POB or prevent DB, could not be analyzed. The procedure time of PuraStat and clipping were calculated using movies; thus, we could not add the time for inserting them through the working channel. Dietary restriction was performed on the day of CSP though it's necessity for CSP was considered controversial.^27^ Authors except one doctor (Y.I) in other institutions work or worked in our university during a study period, and the doctor (Y.I.) helped to make a study design.

## Conclusion

PuraStat was effective for hemostasis of POB and prevention of DB of colorectal CSP in high‐risk cases requiring continuous anticoagulation. Our study suggested that a hemostatic agent can be an alternative method of clipping.

## Supporting information


**Video S1.** POB of CSP treated with PuraStat. A 73‐year‐old man with edoxaban received cold snare polypectomy for multiple polyps using PuraStat for POB and prevention of DB.

## Data Availability

Patient data used to support the findings of this study are available from the corresponding author upon request. However, some of them are restricted by the institutional review board of Kyoto Prefectural University of Medicine.
